# Supplementary material for: Measuring corporate Paris Compliance using a strict science-based approach
Source: Nat Commun. 2022 Aug 10;13:4441. doi: 10.1038/s41467-022-31143-4 (PMC9365807; doi:10.1038/s41467-022-31143-4)
Supplement: Supplementary file 1 — Supplementary Information [file 41467_2022_31143_MOESM1_ESM.pdf]

Supplementary Information

Contrasting the PCP with the TPI and ACT

We here contrast the PCP with the approach by the TPI and ACT, which we demonstrate in Supplementary Figure 1.

The TPI, established in 2017, uses the International Energy Agency sectoral intensity pathway (SI_y), rather than firm-specific carbon intensity (CI_y) proposed by the SDA (Figure 1a). This contrasts clearly with the SDA methodology which uses initial company intensity and projected market share to derive company-specific emissions-intensity trajectories. It therefore also does not adjust the decarbonisation pathway for changes in actual vs. projected company variables. Further, a limitation of the TPI is that it does not provide a re-alignment company-specific decarbonisation trajectory that accounts for the action deficit since the base year. This is also summarized in Supplementary Table 1.

The ACT methodology, developed in 2019, defines benchmarks based on a company's carbon intensity and market share. The ACT is a voluntary initiative of the UNFCCC secretariat Global Climate Agenda supporting corporate climate accountability. It develops sectoral methodologies through a multi-stakeholder process, as an accountability framework to support companies with delivering low carbon transition strategies and actions aligned with the Paris Agreement mitigation goal. As such it is more aligned with the spirit of "bottom-up" governance than the top-down accountability proposed by the Paris agreement goals. It is also a highly flexible approach that can be "plugged in" to a variety of scenarios.

Given its design and purpose it is somewhat unfair to critique the ACT approach based on the one-dimensional warming goals of the Paris Agreement. This is however the key focus of the PCP approach and in this regard the ACT approach presents some problems. For instance, the ACT defines a "previous" benchmark (set at five years before the reporting year) and a "current" benchmark (the reporting year), but by doing so does not account for performance outside of the scope of this 5-year interval. Even if the ACT were to evaluate all companies' performance since 2014 using IEA B2DS (as illustrated for the company in Figure 1b), their "previous" benchmark does not adjust for discrepancies between the actual and projected market share. Its "current benchmark" metric does not account for any emissions performance deficits incurred since the base year. Therefore, the "current" benchmark is not adjusted to account for failure to follow a defined emissions-intensity pathway, a company's original allocated carbon budget would be exceeded if this new benchmark were followed by the company. This is demonstrated in Supplementary Figure 2 below. Further, if companies followed the "current benchmark" by the ACT, i.e. a revised carbon intensity decarbonisation pathway, the carbon budget would be exceeded by nine companies between 1.2 to 1.75 times in the year 2050 (Supplementary Figure 3).

Metrics of the TPI and ACT

Both the TPI and ACT also provide metrics to enable companies to gauge their progress towards their long-term goals. The TPI is designed to require only publicly available data and uses a simple binary carbon performance metric (aligned/not aligned) without quantifying the level of (mis-) alignment. There is no stipulation of what the company needs to do to re-align its trajectory to compensate for any deficits to date. The ACT uses a set of quantitative performance indicators to compare the company's actual and future carbon intensity pathway to their benchmark trajectory. We provide a comparison of our metrics with the TPI and ACT metrics here.

Table A1 provides an overview of the Metrics used by the TPI and ACT, including some key limitations we have identified with each. For the ACT methodology, we evaluated both the ACT framework (for all sectors) [1] and the ACT Sector Methodology Electric Utilities [2]. We selected the indicators that do not require a decarbonisation target to be set by the company, and compare the following metrics for evaluating transition performance: the "action gap", which measures the gap between the company's actual and future carbon intensity to the "previous" benchmark (ACT Framework, Supplementary Figure 5); the "transition ratio", which compares a firm's recent rate of decarbonisation to its required decarbonisation pathway; the "locked-in-emissions", the planned emissions in the next 15 years vs the emissions consistent with the benchmark; and the "future-emissions gap", which measured the alignment of the carbon intensity in 5 years compared to the benchmark. How these differ from the PCP approach is demonstrated in Supplementary Figure 6 and Supplementary Table 3.

Metric 1 is based on companies' emission exceedance and indicates the extent to which a company's current actions are in line with their $PCP_{emissions}$. It is similar to the "action gap" of ACT (ACT Framework) but we quantify the gap between the actual emissions and the $PCP_{emissions}$, and thus account for previous inaction and actual market share to date. Note that the use of absolute emissions is actually acknowledged by the ACT [3]: "Absolute GHG emissions over time are the most relevant measure of emissions performance for assessing a company's contribution to global warming".

Metric 2 provides several indicators of the potential for stranding of existing assets. The sooner a company hits is carbon budget (Metric 2a and b), the faster the company has to decarbonise to operate consistent with staying in their carbon budget, (Metric 2c), and the more it is exposed to the risks associated with transition-driving forces, policies, technologies and markets. The ACT Trend in Future Emission Intensity provides similar information although as explained in Table A1 can be misleading when a consistent baseline is not used.

Metric 3 measures the difference in decarbonisation rates between the "base" $PCP_{intensity}$ and the "re-alignment" $PCP_{intensity}$ and indicates the depth of decarbonisation required for a company to be Paris-Compliant.

We summarize in Supplementary Table 2 the advantages of our metrics over that of the TPI and ACT. Supplementary Table 3 explains the TPI and ACT's metrics, and its limitations, in more detail.

Additional metrics under a "maximum action case" for the 10 Electric Utility companies

For the Australian utility sector we have a rich dataset, and can compute a "maximum action" scenario, which we have defined in the main manuscript as a scenario "where the company continues to operate its assets until the expected closure date and then replaces their assets with a zero-carbon alternative". The results for metric 2b and 2c under a maximum pathway are displayed below.

Company scores

The company scores for Fig 2, Fig 3, Supp Fig 3 and Supp Fig 4 are available in Supplementary Table 4.

References

1. *ACT Framework* (ACT, accessed 21 August 2020); https://actinitiative.org/wp-content/uploads/pdfs/ACT-FRAMEWORK-Eng-2019-04-09.pdf
2. *ACT Electricity* (ACT, accessed 21 August 2020); https://actinitiative.org/resources-2/
3. *ACT Framework* (ACT, accessed 21 August 2020), p.25; https://actinitiative.org/wp-content/uploads/pdfs/ACT-FRAMEWORK-Eng-2019-04-09.pdf
4. Dietz, S. et al. An assessment of climate action by high-carbon global corporations. *Nat. Clim. Change* **8**, 1072-1075 (2018).

Supplementary figures

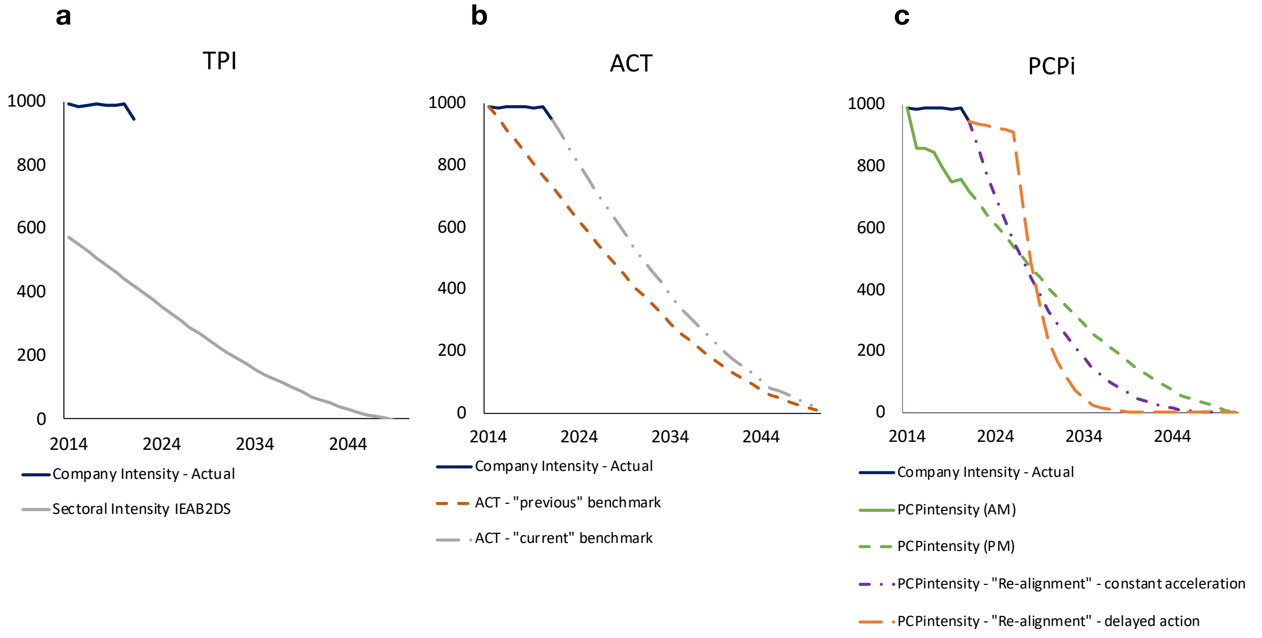

Supplementary Fig. 1 | A comparison of the different approaches that use the SDA allocation method, including that of the TPI, ACT and our PCP approach, each evaluating the transition performance of the same example company (using data until 2019). All panels include the companies' actual carbon intensity since 2014 (black line). Panel a) shows that the Transition Pathway Initiative maps a company's carbon intensity against the sectoral intensity as set out by the IEA B2DS. Panel b) shows that the Assessing low-Carbon Transitions (ACT) initiative uses the SDA to set a "previous" benchmark pathway (dashed orange line), set 5 years prior using projected market (PM) share, and a "current" benchmark pathway (grey dash-dot-dot line) based on current (2019) carbon intensity and projected market share. Panel c) illustrates our methodology showing an "base" Paris-Compliant intensity Pathway (PCP_i) that uses the SDA based on a company's initial intensity (2014) and actual market share (green line) and projected market share (dashed green line). It also shows two possible "re-alignment" $PCP_{intensity}$'s the company could follow to stay within its carbon budget: a $PCP_{intensity}$ featuring a constant "accelerated" decarbonisation rate which commences immediately (dotted orange line); and a $PCP_{intensity}$ and a PCP which follows a "maximum action pathway" for the next 5 years, followed by a new accelerated decarbonisation rate (long-dashed orange line).

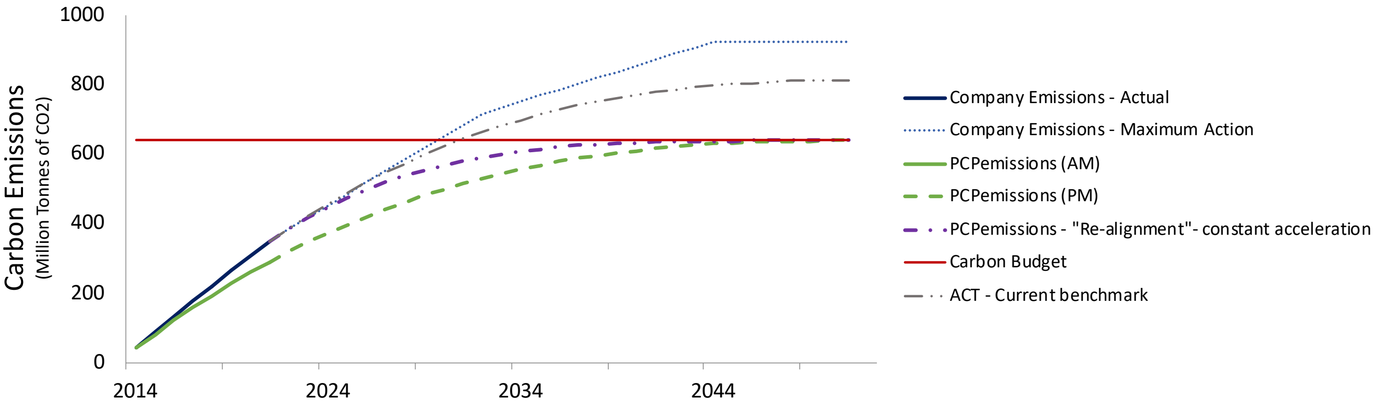

Supplementary Fig. 2 | Comparison of the $PCP_{emissions}$ with the ACT methodology applied to the same example company's emissions. This figure is similar to Figure 2 in the main text, but includes the cumulative emission pathway if the company followed a pathway that is eligible under the Assessing low-Carbon Transitions as illustrated in Supplementary Figure 1b.

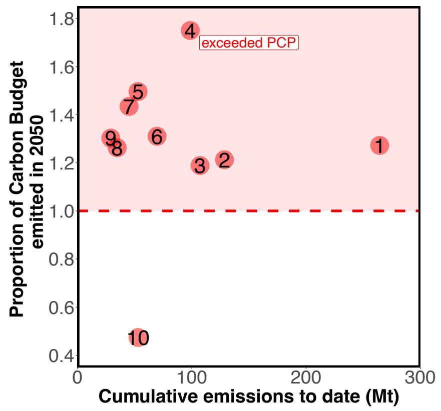

Supplementary Fig. 3 | Transition performance metric 2c if the ten largest energy generators in Australia follow a pathway eligible under the ACT. This figure demonstrates the proportion of the carbon budget emitted in 2050 if the companies followed an emission reduction pathway as eligible under the Assessing low-Carbon Transitions, where the base year of the company is set in 2021, rather than at the start year of the underlying decarbonisation pathway, which is 2014 (IEA B2DS).

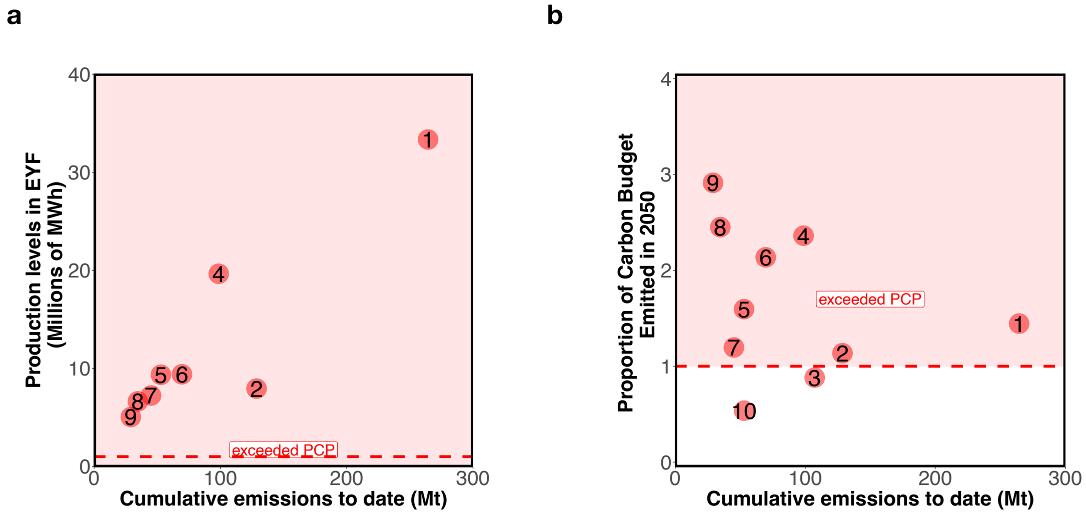

Supplementary Fig 4 | Transition performance metrics for the ten largest producing energy generators in Australia, for metric 2b and 2c under a "maximum action case". Panel a) shows the projected production capacity in the EYF that produces a greater than zero emission intensity (metric 2b), **b)** proportion of the company carbon budget emitted in the year the company's $PCP_{intensity}$ must become net-zero (around 2050 for the electric utilities sector) (metric 2c).

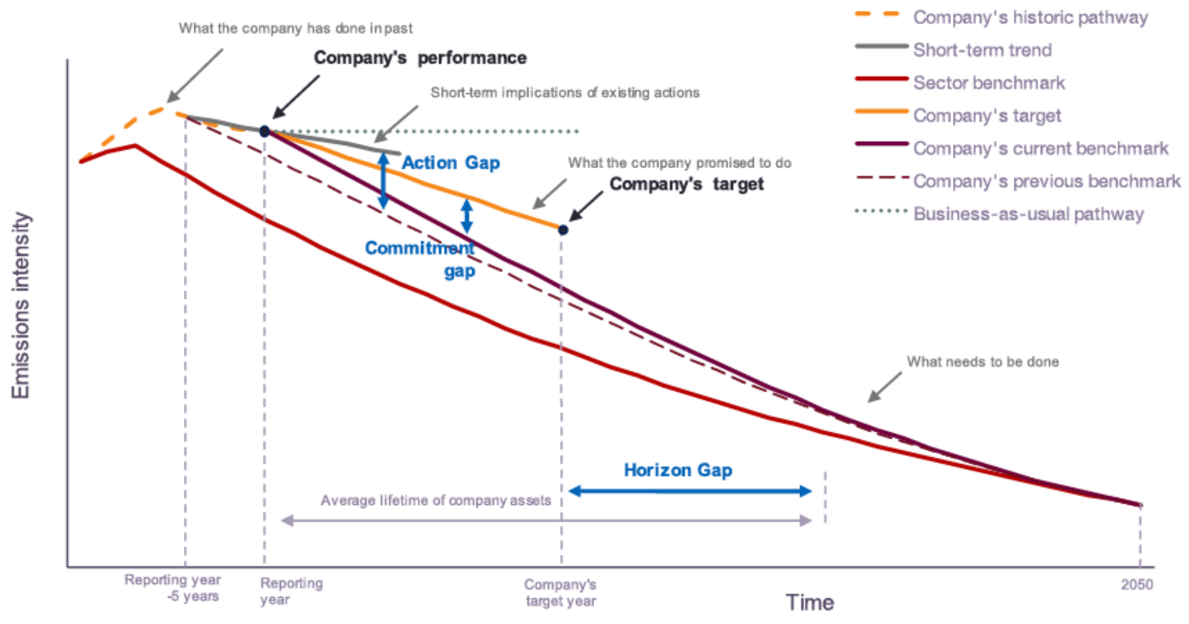

Supplementary Figure 5: ACT metrics. This figure shows the graphical depiction of the ACT metrics as presented in their ACT Framework 2019 [1].

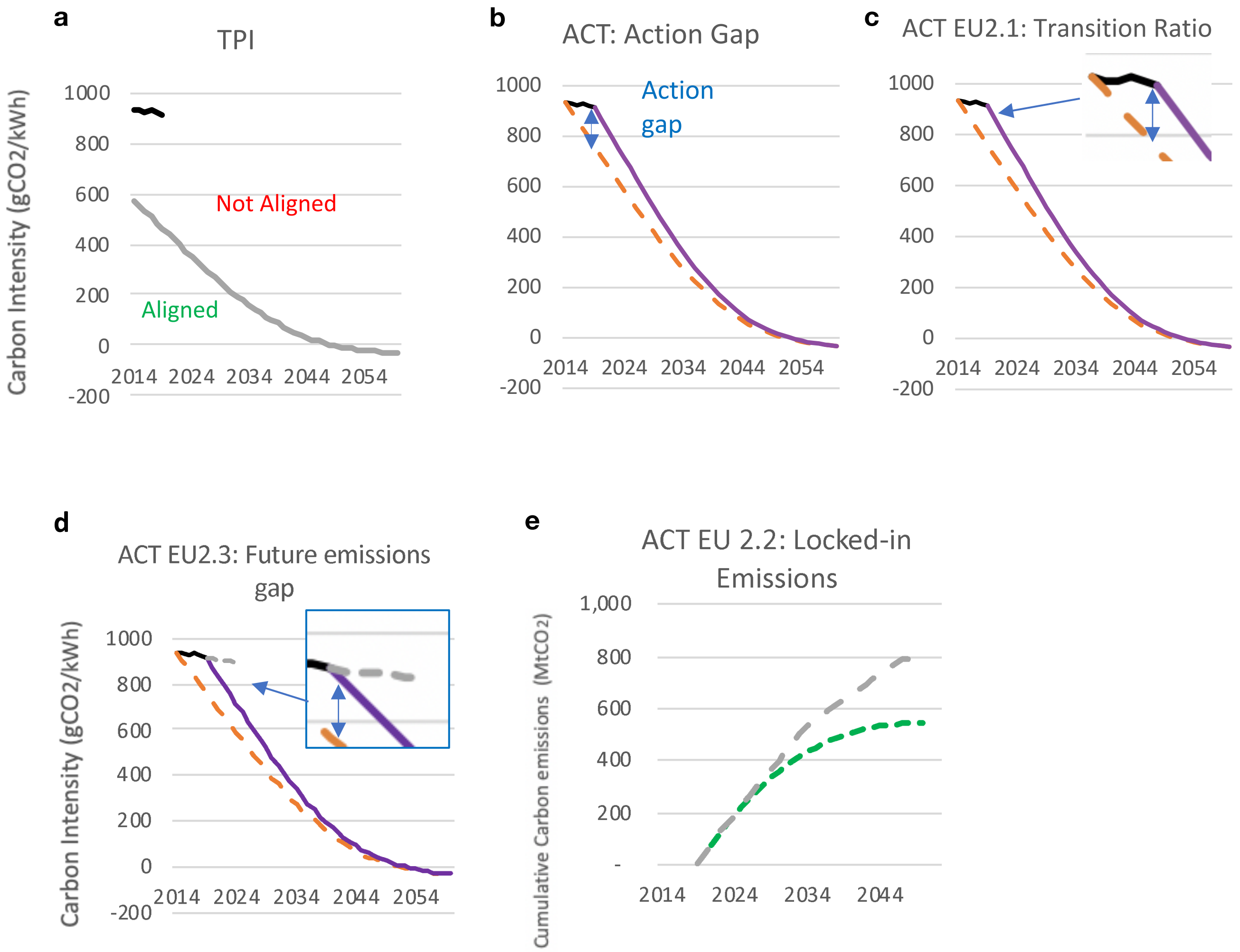

Supplementary Figure 6 | TPI and ACT quantitative metrics for evaluating transition performance for AGL in 2019, the company we used for Figure 2 in the main text. This figure demonstrated the different metrics used by the TPI and ACT to evaluate a company's performance in meeting climate goals. The metrics would show the following: **a)** TPI: The company is *not aligned*. **b)** ACT: Action Gap. The company currently has an action gap (it is unclear from the ACT methodology documentation how to calculate this metric), **c)** ACT EU 2.1 Transition Ratio: The transition ratio for the company would be $\frac{CR'_g}{CB'_g} = \frac{-4.15}{-42.17} = 0.098\ or\ 9.8\%$. If the company would continue its recent (-5yr) trend, the ratio is only around 10% of what is required if it were to follow the benchmark for the next 5 years, **d)** ACT EU 2.2 Locked-in Ratio: assuming business as usual, the default Locked-in Emissions score for the company is: $r_{LB}(t) = \frac{L_g(t)}{B_g(t)} = \frac{538}{434}$ =1.24, giving a score of: $\frac{1.5-1.24}{0.5} = 0.52$ or 52%. (note that this metric does not have an assumption on whether the company will replace its decommissioned assets with a lower emission mix or with net-zero emission, we therefore used an assumption which we refer as the RET – maximum action scenario, see Methods), **e)** ACT EU 2.3 Future emissions gap: The company has a future emissions gap of $\frac{A_g-CB_g}{BAU_g-CB_g} = \frac{899-751}{916-751} = 0.89\ or\ 89\%$, Giving a score of $1 - Future\ emissions\ action\ gap = 1 - 0.89 = 0.11\ or\ 11\%$.

Supplementary tables

	TPI	ACT	PCP
Condition 1: Underlying pathway consistent with Paris Agreement “well-below” 2ºC	✓	✓	✓
Condition 2: Base year consistent with underlying pathway and 2015 or prior	✓	✗	✓
Operationalisation requirement 1: “Base” PCP and carbon budget adjusts yearly for changes in actual vs projected company variables (e.g. market share for SDA or gross profit for CSO)	✗	✗	✓
Operationalisation requirement 1: Re-alignment pathway is defined that corrects for action deficit, so that the company remains within its carbon budget	✗	✗	✓

Supplementary Table 1. Summary of different approaches to defining and operationalising a PCP. This table shows the different approaches by the TPI, ACT and PCP of defining benchmarks.

Supplementary Table 2 | Comparison of ACT, TPI and PCP metrics for measuring transition performance.

Metrics	TPI	ACT	PCP Metrics
Performance to date since common base year (using absolute emissions)	✗ Binary: Aligned/Not aligned, based on intensity pathway only	✗ Action gap, based on intensity pathway only, no common base year used.<br><br>✗ EU 2.1 Transition ratio, based on intensity pathway only, no common base year used.	✓ Metric 1: Cumulative emissions to date compared to $PCP_{emissions}$ since common base year.
Implications of continuing business as usual.	-	✗ EU 2.2 Locked-in emissions: emissions since common base year are ignored.<br><br>✗ EU 2.3: Future Emissions Gap: based on intensity pathway only, benchmark not adjusted for previous performance.	✓ Metric 2a: Estimated Year to Finish (EYF) the Company's Carbon Budget.<br>✓ Metrics 2b: Production with non-net zero carbon intensity in place in EYF.<br>✓ Metrics 2c: Carbon Budget exceedance at around 2050, when the company's $PCP_{intensity}$ ought to reach zero.
Depth of decarbonisation required to be climate-safe.		-	✓ Metric 3: "extra" decarbonisation rate required to stay within the carbon budget, compared to if the company had followed its $PCP_{intensity}$ from the base year.

Supplementary Table 3 – Extended table on TPI and ACT quantitative benchmarks and metrics on transition performance.

<table>
<tr><td>Framework
TPI</td><td>Benchmarks
a. Sectoral Emission intensity (below 2 degrees)
b. Sectoral Emission intensity (2 degrees)
c. Emission intensity (NDCs/Paris Pledges)</td></tr>
<tr><td colspan="2">Metrics
If the companies' carbon intensity is less than a) then Aligned, otherwise Not Aligned. Designed for simplicity to enable the use of publicly available data (Dietz et al 2018b).

<u>Limitations:</u> Provides no metrics indicating the level of misalignment with net zero goals, nor guidance on how to re-align.

All other metrics used for TPI are qualitative.

An example of how the TPI would rate our anonymous company used for Figure 1 and 2 in the main text can be found in Supplementary Figure 6.</td></tr>
<tr><td>Framework
ACT Framework (2019) [1]</td><td>Benchmarks
Follows SBTi:
- convergence of carbon intensity (SDA)
- compression of carbon intensity
- contraction of absolute emissions</td></tr>
<tr><td colspan="2">Metrics

See Supplementary Table 1 for a depiction of the ACT's metrics as presented in their ACT Framework 2019 [1].

1. Commitment gap
<u>Description by ACT</u>: The difference between what needs to be done and what the company says it will do.

<u>Limitations</u>: Uses an arbitrarily set time frame (reporting year and reporting year – 5 years) that does not consider (mis) performance since a common base year. Needs company to set reporting year and targets for this metric to be used, otherwise it is the same as the action gap.

2. Horizon gap</td></tr>
</table>

Description by ACT: The difference between the average lifetime of the company's production assets (particularly carbon intensive) and the time horizon of its commitments.

Limitations: Needs company to set target to a certain date for this metric to be used. Does not measure climate performance.

3. Action gap (note: this metric was removed in the updated ACT Generic Methodology 2021)
Description by ACT: The difference between what the company has done in the past plus what it is doing now and what needs be done.

Limitations: Arbitrarily set time frame that does not consider performance (or lack thereof) based on a common base year. The action gap is determined by comparing the company's trend against their "previous' benchmark. The "previous" benchmark fails to account for a company diverging from a company's intended or projected market share to date. Carbon intensity is difficult to interpret and does not give a clear picture of its over-emissions to date. We propose Metric 1 (see methods) as a metric that overcomes all these shortcomings.
* The Action Gap metric has been removed from the ACT general methodology in the April 2021 update.

**Framework**	**Benchmark**
ACT<br>Sector<br>Methodology<br>Electricity (2019)<br>[2]	$CB_G =$ (gCO2/kWh) using the SDA methodology and IEA 2DS (not B2DS) (p.48), using "initial" intensity and market share (projection) is based on regional activity (projection) - where "initial" refers to the reporting year.<br>Limitations: Using the reporting year as the base year is problematic, as discussed, and even if this year is consistent with the IEA pathway, it is unclear how the benchmark is adjusted to account for performance to date, especially how the benchmark needs to be adjusted to make up for any deficits.

Metrics

EU 1.1 – Alignment of Scope 1+2 emissions reductions targets (future)

Description by ACT: Measures the difference between the company's target ($T_{S1,(2)}$) and the company benchmark (CB_G) 5 years after the reporting year.

Based on the figure in the ACT framework (above) we assume that CB_G is set at the current reporting year using a projection of market share.

The commitment gap is calculated as follows:

$$Commitment\ gap = \frac{T_{s1} - CB_g}{BAU_g - CB_g}$$

$Score = 1 - commitment\ gap$ (expressed as %)

If $T_{s1} = CB_g$, then the maximum score is achieved.

Limitations: Uses an arbitrarily set time frame (reporting year and reporting year – 5 years) that does not consider (mis) performance since a common base year. Needs company to set reporting year and targets for this metric to be used, otherwise it is the same as the action gap measured in EU 2.3.

EU 1.2 - Time horizon of targets (future)

See Horizon Gap above in ACT Framework (2019)

EU 1.3 – Achievement of previous targets (past and present)

Qualitative and not included in performance indicators.

EU 2.1 – Trend in past emissions intensity (past and present)

Description by ACT: A measure of the alignment of the company's recent emissions intensity trend with that of its decarbonization pathway.

Calculated by the transition ratio:

$$r_{s1} = \frac{CR'_G}{CB'_g}$$

Where, CR'_G represents the company's recent (reporting year minus 5 years) emissions intensity trend gradient.

CB'_G represents the company's decarbonisation pathway trend gradient (reporting year plus 5 years). However, we assume CB_G is set at the current reporting year using a projection of market share, consistent with EU1.1.

Limitations: Uses an arbitrarily set time frame (reporting year ± 5 years) that does not consider (mis) performance since a common base year. It allows the company to keep using a "new" benchmark pathway, that whilst steeper, does not account for deficits since the base year. It is also based on intensity only, which does not provide an accurate representation of the performance to date. Our proposed Metric 1 (see main text and methods) addresses these shortcomings.

EU 2.2 Locked-in emissions (future)

Description by ACT: A measure of the company's cumulative generation emissions from the reporting up to 2050 from installed and planned power plants. Analysed as follows: Ratio between the company's installed and planned emissions for the 15 years after the reporting year and the emissions budget entailed by the company's carbon budget over the same number of years.

Company's locked-in carbon commitments at time t are calculated as follows:

$$L_g(t) = \int_{reporting\ year}^{t} A_g * CA_g$$

where A_g is the generation activity and CA_g the company's intensity pathway.

The company's carbon budget at time t is calculated as follows:

$$B_g(t) = \int_{reporting\ year}^{t} A_g * CB_g$$

where A_g is the generation activity and CB_g the company's benchmark pathway.

The locked-in ratio is then calculated as follows:

$$r_{LB}(t) = \frac{L_g(t)}{B_g(t)}$$

Maximum score is 100%.
If r_{LB} > 1.5, a minimum score of 0% is assigned.
If 1< r_{LB} < 1.5, then a score of 1.5 - r_{LB} divided by 50% is assigned.

Limitations: Company carbon budget relies on CB_g, which is the company benchmark unadjusted for historical performance. The default value for is based on the next 15. Any performance to since the base year is not accounted for. We propose Metrics 2a, b, and c (see methods) as an alternative that overcomes these shortcomings.

EU 2.3 Trend in future emission intensity (future)
Description by ACT: Metric to identify the gap in 5 years after the reporting year between the company's performance and the decarbonization pathway as a percentage, which is expressed as the company's 'action gap'.

$$Future\ emissions\ action\ gap = \frac{A_g - CB_g}{BAU_g - CB_g}$$

$$Score = 1 - Future\ emissions\ action\ gap$$

in which A_g represents the generation weighted-average plant emission intensity in 5 years, CB_g represents the company-specific decarbonisation pathway emission intensity in 5 years, BAU_g represents the reporting year's emission intensity.
If $A_g - CB_g$ is zero the maximum score will be achieved.

Limitations: see "Action Gap" above.

EU3 -EU9 do not use benchmarks for emission reduction levels

Supplementary Table 4 – Company scores. This table shows the scores as depicted in Figure 2 and 3 of the main text.

Figure 3a-c (Electric Utilities)

Firm	Name	Cumulative Emissions	Metric 1 (Fig 3a)	Metric 2A (Fig 3b)	Metric 3 (Fig 3c)
1	AGL	264	1.20	2030	1.77
2	EnergyAustralia	129	1.02	2033	1.09
3	Origin	107	1.10	-	1.17
4	Stanwell	99	1.54	2025	4.02
5	CSEnergy	53	1.34	2027	2.59
6	Alinta	70	1.05	2029	1.21
7	Delta	45	1.41	2026	2.54
8	Milmerran	35	1.16	2029	1.73
9	Callide	29	1.41	2026	2.16
10	Engie	53	0.50	-	0.00

Figure 3d-f (Cement)

Firm	Name	Cumulative Emissions	Metric 1 (Fig 3d)	Metric 2A (Fig3e)	Metric 3 (Fig 3f)
1	HEIDELBERGCEMENT (XET)	391	1.36	2035	2.28
2	ACC	95	1.07	2046	1.01
3	AMBUJA CEMENTS	85	1.04	2043	1.15
4	ULTRATECH CEMENT	234	1.22	2038	2.02
5	SHREE CEMENT	73	1.30	2039	2.33
6	CRH (LON)	149	2.08	2029	6.55
7	HOLCIM	754	1.19	2044	1.40
8	ASIA CEMENT	24	1.02	2039	1.25
9	SIAM CEMENT	130	1.05	2042	1.13
10	CEMEX CPO	252	1.07	2040	1.23

Supp Fig 3, 4A and 4B

Firm	Name	Cumulative Emissions	ACT 2050 (SuppFig3)	Metric 2B (SuppFig4a)	Metric 2C (SuppFig4b)
1	AGL	264	1.27	33.34	1.44
2	EnergyAustralia	129	1.21	7.94	1.14
3	Origin	107	1.19		0.88
4	Stanwell	99	1.75	19.65	2.36
5	CSEnergy	53	1.50	9.35	1.60
6	Alinta	70	1.31	9.40	2.14
7	Delta	45	1.43	7.24	1.20
8	Milmerran	35	1.26	6.64	2.45
9	Callide	29	1.30	5.05	2.91
10	Engie	53	0.47		0.54
