# Supplementary material for: Measuring corporate Paris Compliance using a strict science-based approach
Source: Nat Commun. 2022 Aug 10;13:4441. doi: 10.1038/s41467-022-31143-4 (PMC9365807; doi:10.1038/s41467-022-31143-4)
Supplement: Supplementary file 4 — Supplementary Data [file 41467_2022_31143_MOESM4_ESM.pdf]

```
#libraries


library(ggplot2)
library(hrbrthemes)
library(scales)
library(readxl)
library(ggrepel)
library(ggthemes)
library(reshape2)
library(tidyr)
library(gridExtra)

#make sure to make a folder on your desktop that is named "Figure3" and contains the data files

setwd("~/Desktop/Figure3")

#spreadsheets in the Figure3 folder should be structured in a certain way, see the spreadsheet structures

Figure3a_EU <- read_excel("FigureData_ElectricUtilities.xlsx",sheet = "Metric1")
Figure3b_EU <- read_excel("FigureData_ElectricUtilities.xlsx",sheet = "Metric2A")
Figure3c_EU <- read_excel("FigureData_ElectricUtilities.xlsx",sheet = "Metric3")

Figure3d_CE <- read_excel("FigureData_Cement.xlsx",sheet = "Metric1")
Figure3e_CE <- read_excel("FigureData_Cement.xlsx",sheet = "Metric2A")
Figure3f_CE <- read_excel("FigureData_Cement.xlsx",sheet = "Metric3")

SuppFigure3_EU <- read_excel("FigureData_ElectricUtilities.xlsx",sheet = "SuppFig3")
SuppFigure4a_EU <- read_excel("FigureData_ElectricUtilities.xlsx",sheet = "SuppFig4_M2B")
SuppFigure4b_EU <- read_excel("FigureData_ElectricUtilities.xlsx",sheet = "SuppFig4_M2C")

########################################################################
########################################################################
######################


####################Figure 3a, d METRIC 1 (March 29 2022)
#####################################################

    ############ELECTRIC UTILITIES######################

      plot_figure3a <- ggplot(Figure3a_EU, aes( x= Cumulative_Emissions, y = Metric1))+
       geom_point(fill="red", color="red", shape=16, alpha=0.5, size=14)+
       annotate("rect", xmin = -Inf, xmax = Inf, ymin = 1, ymax = Inf, alpha = 0.1, fill = "red")+
       geom_text(aes(label=rownames(Figure3a_EU)), size=12)+
       labs(size = "        MWh
          (2019 Millions)",title="",
          x="Cumulative emissions to date (Mt)",
          y="Proportion of Paris Compliant
 Pathway emitted in 2021")+
       theme( legend.background = element_rect(fill = "white", color = "black"),
           axis.title=element_text(size=30,face="bold"),
           legend.text=element_text(size=20),
           axis.text=element_text(size=30),
           legend.box.background = element_rect(colour = "black"),
           legend.position = "right",
           text=element_text(size=10),
           plot.title = element_text(hjust = .5),
           axis.line = element_line(colour = "black"),
```

```
        panel.border = element_rect(colour = "black", fill=NA, size=3),
        panel.background = element_blank(),
        plot.margin = unit(c(1,1,1,1),"cm"))+
  ggplot2::annotate("label", 150,1.7, label="exceeded PCP", color="red", size=8)+
  geom_hline(yintercept=1, color="red", linetype="dashed", size=2)+
  scale_size_continuous(range=c(5,35))+
  scale_x_continuous(breaks=seq(0,300,100), limits=c(0,300), expand = expansion(mult = 0, 0.05))+
  scale_y_continuous(breaks=seq(0.4,1.8,0.2), limits=c(0.4,1.8), expand = expansion(mult = 0, 0.05))

plot_figure3a

############CEMENT#######################

plot_figure3d <- ggplot(Figure3d_CE, aes( x= Cumulative_Emissions, y = Metric1))+
  geom_point(fill="blue", color="blue", shape=17, alpha=0.5, size=14)+
  annotate("rect", xmin = -Inf, xmax = Inf, ymin = 1, ymax = Inf, alpha = .1, fill = "red")+
  geom_text(aes(label=rownames(Figure3d_CE)), size=12)+
  labs(size = "        MWh
     (2019 Millions)",title="",
     x="Cumulative emissions to date (Mt)",
     y="Proportion of Paris Compliant
Pathway emitted in 2020")+
  theme( legend.background = element_rect(fill = "white", color = "black"),
        axis.title=element_text(size=30,face="bold"),
        legend.text=element_text(size=20),
        axis.text=element_text(size=30),
        legend.box.background = element_rect(colour = "black"),
        legend.position = "right",
        text=element_text(size=15),
        plot.title = element_text(hjust = 0.5),
        axis.line = element_line(colour = "black"),
        panel.border = element_rect(colour = "black", fill=NA, size=3),
        panel.background = element_blank(),
        plot.margin = unit(c(1,1,1,1),"cm"))+
  ggplot2::annotate("label", 150,1.6, label="exceeded PCP", color="red", size=8)+
  geom_hline(yintercept=1, color="red", linetype="dashed", size=2)+
  scale_size_continuous(range=c(5,35))+
  scale_x_continuous(breaks=seq(0,900,300), limits=c(0,900), expand = expansion(mult = 0, 0.05))+
  scale_y_continuous(breaks=seq(0.4,2.4,0.2), limits=c(0.4,2.4), expand = expansion(mult = 0, 0.05))

plot_figure3d


###################Figure 3b, e METRIC 2(a) (March 29 2022)
#####################################################

############# Figure 3b ELECTRIC UTILITIES #######################
figure3b <- ggplot(Figure3b_EU, aes(x = Cumulative_Emissions, y = EYF))+
  geom_point(fill="red", color="red", shape=16, alpha=0.5, size=14)+
  annotate("rect", xmin = -Inf, xmax = Inf, ymin = 2015, ymax = 2050, alpha = .1, fill = "red")+
  geom_text(aes(label=rownames(Figure3b_EU)), size=12)+
  labs(size ="",title="",
     x="Cumulative emissions to date (Mt)",
     y="Estimated Year to Finish (EYF)")+
  theme( legend.background = element_rect(fill = "white", color = "black"),
        axis.title=element_text(size=30,face="bold"),
        legend.text=element_text(size=20),
        axis.text=element_text(size=30),
        legend.box.background = element_rect(colour = "black"),
```

```
        legend.position = "right",
        text=element_text(size=15),
        plot.title = element_text(hjust = 0.5),
        axis.line = element_line(colour = "black"),
        panel.border = element_rect(colour = "black", fill=NA, size=3),
        panel.background = element_blank(),
        plot.margin = unit(c(1,1,1,1),"cm"))+
    geom_hline(yintercept=2060, color="red", linetype="dashed", size=2)+
    scale_size_continuous(range=c(5,35))+
    scale_y_continuous(breaks=seq(2015,2055,10), limits=c(2015,2060), expand = expansion(mult =
0, 0.05))+
    scale_x_continuous(breaks=seq(0,300,100), limits=c(0,300), expand = expansion(mult = 0, 0.05))

figure3b


############# Figure 3e CEMENT#######################

figure3e <- ggplot(Figure3e_CE, aes(x = Cumulative_Emissions, y = EYF))+
  geom_point(fill="blue", color="blue", shape=17, alpha=0.5, size=14)+
  annotate("rect", xmin = -Inf, xmax = Inf, ymin = 2015, ymax = 2060, alpha = .1, fill = "red")+
  geom_text(aes(label=rownames(Figure3b_EU)), size=12)+
  labs(size ="",title="",
     x="Cumulative emissions to date (Mt)",
     y="Estimated Year to Finish (EYF)")+
  theme( legend.background = element_rect(fill = "white", color = "black"),
        axis.title=element_text(size=30,face="bold"),
        legend.text=element_text(size=20),
        axis.text=element_text(size=30),
        legend.box.background = element_rect(colour = "black"),
        legend.position = "right",
        text=element_text(size=15),
        plot.title = element_text(hjust = 0.5),
        axis.line = element_line(colour = "black"),
        panel.border = element_rect(colour = "black", fill=NA, size=3),
        panel.background = element_blank(),
        plot.margin = unit(c(1,1,1,1),"cm"))+
  geom_hline(yintercept=2060, color="red", linetype="dashed", size=2)+
  scale_size_continuous(range=c(5,35))+
  scale_y_continuous(breaks=seq(2015,2065,10), limits=c(2015,2070), expand = expansion(mult = 0,
0.1))+
  scale_x_continuous(breaks=seq(0,900,300), limits=c(0,900), expand = expansion(mult = 0, 0.1))

figure3e


###################Figure 3c, f METRIC 2(a) (March 29 2022)
#####################################################

#############  Figure 3c ELECTRIC UTILITIES   #######################

figure3c <- ggplot(Figure3c_EU, aes(x=Cumulative_Emissions, y=Metric3))+
  geom_point(fill="red", color="red", shape=16, alpha=0.5, size=14)+
  geom_text(aes(label=rownames(Figure3c_EU)), size=12)+
  labs(title="",
     x="Cumulative emissions to date (Mt)",
     y="Required decarbonisation rate multiplier
     to meet carbon budget")+
  theme(axis.ticks.x = element_blank(),
```

```
      axis.ticks.y = element_blank(),
      legend.background = element_rect(fill = "white", color = "black"),
      axis.title=element_text(size=30,face="bold"),
      legend.text=element_text(size=20),
      axis.text=element_text(size=30),
      legend.box.background = element_rect(colour = "black"),
      legend.position = "right",
      text=element_text(size=20),
      plot.title = element_text(hjust = 0.5),
      axis.line = element_line(colour = "black"),
      panel.border = element_rect(colour = "black", fill=NA, size=3),
      panel.background = element_blank(),
      plot.margin = unit(c(1,1,1,1),"cm"))+
  scale_y_continuous(breaks=seq(0,6, 2), limits=c(0, 6), expand = expansion(mult = 0, 0.1))+
  scale_x_continuous(breaks=seq(0, 300, 100), limits=c(0, 300), expand = expansion(mult = 0, 0.1))

figure3c

figure3f <- ggplot(Figure3f_CE, aes(x=Cumulative_Emissions, y=Metric3))+
  geom_point(fill="blue", color="blue", shape=17, alpha=0.5, size=14)+
  geom_text(aes(label=rownames(Figure3f_CE)), size=12)+
  labs(title="",
     x="Cumulative emissions to date (Mt)",
     y="Required decarbonisation rate multiplier
     to meet carbon budget")+
  theme(axis.ticks.x = element_blank(),
      axis.ticks.y = element_blank(),
      legend.background = element_rect(fill = "white", color = "black"),
      axis.title=element_text(size=30,face="bold"),
      legend.text=element_text(size=20),
      axis.text=element_text(size=30),
      legend.box.background = element_rect(colour = "black"),
      legend.position = "right",
      text=element_text(size=20),
      plot.title = element_text(hjust = 0.5),
      axis.line = element_line(colour = "black"),
      panel.border = element_rect(colour = "black", fill=NA, size=3),
      panel.background = element_blank(),
      plot.margin = unit(c(1,1,1,1),"cm"))+
  scale_y_continuous(breaks=seq(0,6, 2), limits=c(0, 6), expand = expansion(mult = 0, 0.1))+
  scale_x_continuous(breaks=seq(0, 900, 300), limits=c(0, 900), expand = expansion(mult = 0, 0.1))

figure3f


############### SUPPLEMENTARY FIGURES
##################################################

plot_suppfig3 <- ggplot(SuppFigure3_EU, aes( x= Cumulative_Emissions, y = ACTexceedance))+
  geom_point(fill="red", color="red", shape=16, alpha=0.5, size=14)+
  annotate("rect", xmin = -Inf, xmax = Inf, ymin = 1, ymax = Inf, alpha = 0.1, fill = "red")+
  geom_text(aes(label=rownames(SuppFigure3_EU)), size=12)+
  labs(size = "        MWh
     (2019 Millions)",title="",
     x="Cumulative emissions to date (Mt)",
     y="Proportion of Carbon Budget
emitted in 2050")+
  theme( legend.background = element_rect(fill = "white", color = "black"),
       axis.title=element_text(size=30,face="bold"),
```

```
        legend.text=element_text(size=20),
        axis.text=element_text(size=30),
        legend.box.background = element_rect(colour = "black"),
        legend.position = "right",
        text=element_text(size=10),
        plot.title = element_text(hjust = .5),
        axis.line = element_line(colour = "black"),
        panel.border = element_rect(colour = "black", fill=NA, size=3),
        panel.background = element_blank(),
        plot.margin = unit(c(1,1,1,1),"cm"))+
  ggplot2::annotate("label", 150,1.7, label="exceeded PCP", color="red", size=8)+
  geom_hline(yintercept=1, color="red", linetype="dashed", size=2)+
  scale_size_continuous(range=c(5,35))+
  scale_x_continuous(breaks=seq(0,300,100), limits=c(0,300), expand = expansion(mult = 0, 0.05))+
  scale_y_continuous(breaks=seq(0.4,1.8,0.2), limits=c(0.4,1.8), expand = expansion(mult = 0, 0.05))

plot_suppfig3


plot_suppfig4a <- ggplot(SuppFigure4a_EU, aes( x= Cumulative_Emissions, y = Metric2B))+
  geom_point(fill="red", color="red", shape=16, alpha=0.5, size=14)+
  annotate("rect", xmin = -Inf, xmax = Inf, ymin = 0, ymax = Inf, alpha = 0.1, fill = "red")+
  geom_text(aes(label=rownames(SuppFigure3_EU)), size=12)+
  labs(size = "        MWh
      (2019 Millions)",title="",
      x="Cumulative emissions to date (Mt)",
      y="Production levels in EYF
(Millions of MWh)")+
  theme( legend.background = element_rect(fill = "white", color = "black"),
        axis.title=element_text(size=30,face="bold"),
        legend.text=element_text(size=20),
        axis.text=element_text(size=30),
        legend.box.background = element_rect(colour = "black"),
        legend.position = "right",
        text=element_text(size=10),
        plot.title = element_text(hjust = .5),
        axis.line = element_line(colour = "black"),
        panel.border = element_rect(colour = "black", fill=NA, size=3),
        panel.background = element_blank(),
        plot.margin = unit(c(1,1,1,1),"cm"))+
  ggplot2::annotate("label", 150,1.7, label="exceeded PCP", color="red", size=8)+
  geom_hline(yintercept=1, color="red", linetype="dashed", size=2)+
  scale_size_continuous(range=c(5,35))+
  scale_x_continuous(breaks=seq(0,300,100), limits=c(0,300), expand = expansion(mult = 0, 0.05))+
  scale_y_continuous(breaks=seq(0,40,10), limits=c(0,40), expand = expansion(mult = 0, 0.05))

plot_suppfig4a

plot_suppfig4b <- ggplot(SuppFigure4b_EU, aes( x= Cumulative_Emissions, y = Metric2B))+
  geom_point(fill="red", color="red", shape=16, alpha=0.5, size=14)+
  annotate("rect", xmin = -Inf, xmax = Inf, ymin = 1, ymax = Inf, alpha = 0.1, fill = "red")+
  geom_text(aes(label=rownames(SuppFigure3_EU)), size=12)+
  labs(size = "        MWh
      (2019 Millions)",title="",
      x="Cumulative emissions to date (Mt)",
      y="Proportion of Carbon Budget
Emitted in 2050")+
  theme( legend.background = element_rect(fill = "white", color = "black"),
        axis.title=element_text(size=30,face="bold"),
```

```
        legend.text=element_text(size=20),
        axis.text=element_text(size=30),
        legend.box.background = element_rect(colour = "black"),
        legend.position = "right",
        text=element_text(size=10),
        plot.title = element_text(hjust = .5),
        axis.line = element_line(colour = "black"),
        panel.border = element_rect(colour = "black", fill=NA, size=3),
        panel.background = element_blank(),
        plot.margin = unit(c(1,1,1,1),"cm"))+
  ggplot2::annotate("label", 150,1.7, label="exceeded PCP", color="red", size=8)+
  geom_hline(yintercept=1, color="red", linetype="dashed", size=2)+
  scale_size_continuous(range=c(5,35))+
  scale_x_continuous(breaks=seq(0,300,100), limits=c(0,300), expand = expansion(mult = 0, 0.05))+
  scale_y_continuous(breaks=seq(0,4,1), limits=c(0,4), expand = expansion(mult = 0, 0.05))

plot_suppfig4b
```
